# Transcatheter Aortic Valve Replacement and Concomitant Mitral Regurgitation

**DOI:** 10.3389/fcvm.2018.00074

**Published:** 2018-06-19

**Authors:** Barbara E. Stähli, Markus Reinthaler, David M. Leistner, Ulf Landmesser, Alexander Lauten

**Affiliations:** ^1^Department of Cardiology, Charité - Universitätsmedizin Berlin, Berlin, Germany; ^2^Partner Site Berlin, Deutsches Zentrum für Herz-Kreislaufforschung (DZHK), Berlin, Germany; ^3^Berlin Institute of Health, Berlin, Germany

**Keywords:** transcatheter aortic vave replacement, mitral valve insufficiency, mitral valve repair, aortic stenosis, aortic valve, mitral valve

## Abstract

Mitral regurgitation frequently coexists in patients with severe aortic stenosis. Patients with moderate to severe mitral regurgitation at the time of transcatheter aortic valve replacement are at increased risk of future adverse events. Whether concomitant mitral regurgitation is independently associated with worse outcomes after TAVR remains a matter of debate. The optimal therapeutic strategy in these patients—TAVR with evidence-based heart failure therapy, combined TAVR and transcatheter mitral valve intervention, or staged transcatheter therapies—is ill-defined, and guideline-based recommendations in patients at increased risk for open heart surgery are lacking. Hence, a thorough evaluation of the aortic and mitral valve anatomy and function, along with an in-depth assessment of the patients' baseline risk profile, provides the basis for an individualized treatment approach. The aim of this review is therefore to give an overview of the current literature on mitral regurgitation in TAVR, focusing on different diagnostic and therapeutic strategies and optimal clinical decision making.

## Introduction

Concomitant mitral regurgitation is frequently observed in patients with severe aortic stenosis (1–3). About 20% of patients undergoing transcatheter (TAVR) or surgical (SAVR) aortic valve replacement for severe aortic stenosis have concomitant more than mild mitral regurgitation (1–3). Whether concomitant mitral regurgitation is independently associated with worse outcomes after aortic valve replacement is uncertain (4). A thorough evaluation of the aortic and mitral valve anatomy and function is important in these patients and mainly based on transthoracic and transesophageal echocardiography. An in-depth understanding of the underlying pathophysiological mechanism provides the basis for an individualized treatment approach and optimal procedural planning. Emerging minimally invasive surgical and transcatheter treatment strategies offer novel, less-invasive therapeutic options for combined, staged or hybrid procedures when severe aortic stenosis and mitral regurgitation do coexist, particularly in elderly patients, obviating the need for open heart surgery (5). The treatment of first choice in these patients, however, remains a matter of debate, and guideline-based recommendations are lacking.

The aim of this review is therefore to give an overview of the current literature on mitral regurgitation in TAVR, with particular focus on the different diagnostic and therapeutic strategies available and on the clinical decision-making process in patients at increased surgical risk.

## Assessment of mitral regurgitation in patients with severe aortic stenosis

In mitral regurgitation, besides the grading of the regurgitation severity, identification of the underlying etiology, particularly the distinction between primary and secondary mitral regurgitation, is of great importance to guide therapeutic management. The assessment of the mitral valve apparatus and the type of dysfunction is mainly based on transthoracic and/or transesophageal echocardiography with multimodality imaging used in specific situations (6, 7). Although transthoracic echocardiography is diagnostic in most cases, transesophageal echocardiography complements the assessment when transthoracic image quality is suboptimal and further diagnostic refinement is required (6, 7). Transesophageal echocardiography not only provides additional important information on the etiology of the disease, but also helps to determine the feasibility of dedicated transcatheter mitral valve procedures. Three-dimensional (3D) echocardiography facilitates anatomic and functional interpretation, particularly in patients with complex valvular pathologies (6–9).

Mitral regurgitation may either be primary/degenerative due to abnormalities of the valvular apparatus itself such as mitral valve prolapse, flail leaflets, and chordal rupture, or secondary/functional due to restricted leaflets, mostly caused by left ventricular dilatation and dysfunction in ischemic cardiomyopathy and chronic pressure overload related to aortic stenosis (10). Annular dilatation and left atrial enlargement causing insufficient leaflet closure, e. g., in patients with long-standing atrial fibrillation, may also be an underlying cause. Mixed forms exist when both pathologies overlap. As the mitral valvular apparatus is often calcified in patients with degenerative aortic stenosis, pure secondary mitral regurgitation is unlikely in this context (4).

An integrated approach using qualitative, semi-quantitative, and quantitative echocardiographic parameters allows for a comprehensive assessment of mitral regurgitation (9, 11, 12). Color flow imaging is the most common way to detect mitral regurgitation, with quantification based on the integration of further measures such as vena contracta width, PISA radius, regurgitation volume and effective regurgitant orifice area (EROA) (6). The evaluation of mitral regurgitation in aortic stenosis may, however, be challenging as jet velocity may be increased due to high left ventricular pressures (4). On the other hand, concomitant mitral regurgitation impacts on transvalvular gradient and flow in severe aortic stenosis, which may hamper echocardiographic assessment (13).

A thorough echocardiographic evaluation of the mitral valve apparatus is needed to determine the feasibility of transcatheter mitral valve interventions (14–16). Unfavorable echocardiographic criteria for percutaneous edge-to-edge mitral valve repair include severe leaflet calcifications in the grasping area, rheumatic leaflet thickening, perforated leaflets or clefts, and a mobile length of the posterior mitral valve leaflet of <7 mm, along with insufficient mechanical coaptation in functional (coaptation depth >11 mm, coaptation length <2 mm) and excessive flail gap in degenerative disease (fail gap >10 mm and flail width >15 mm, Table 1) (15–17). A pre-procedural mitral valve area of >4 cm^2^ is recommended in order to reduce the risk of post-procedural mitral valve stenosis (17). Advanced imaging modalities such as multidetector computed tomography (MDCT) complement the assessment of these patients. Besides the evaluation of the aorto-iliacal axis in TAVR patients, MDCT provides important information on the mitral valve apparatus, particularly on annular dimensions, the extent and localization of calcifications, and the spacial relationship to adjacent structures (16, 18, 19).

**Table 1:** Favorable echocardiographic criteria for transcatheter edge-to-edge mitral valve repair with the MitraClip® system.

**Favorable echocardiographic criteria**	**Unfavorable echocardiographic criteria**
Regurgitation located in the midportion of the valve	Rheumatic valve disease
Absence of leaflet calcifications in the grasping area	Leaflet perforation or clefts
Mitral valve area >4 cm2	Mitral stenosis
Posterior leaflet length ≥10 mm	Posterior leaflet length <7 mm
Flail gap <10 mm and flail width <15 mm	
Coaptation depth <11mm and coaptation length >2 mm	

*Adapted from Wunderlich and Siegel (17)*.

## Impact of mitral regurgitation on outcomes in patients with severe aortic stenosis

Patients with aortic stenosis and coexisting moderate to severe mitral regurgitation are known to have a worse clinical risk profile as compared to those without, which is also reflected by higher surgical risk scores (20, 21). They are older, have a higher prevalence of atrial fibrillation and prior myocardial infarction, and poorer left ventricular systolic function (LVEF) (20–22). Whether concomitant mitral regurgitation independently affects outcomes in patients undergoing AVR remains an ongoing matter of debate, particularly whether secondary mitral regurgitation is related with outcomes irrespective of left ventricular dysfunction. While some studies did not observe any association between the presence of mitral regurgitation and adverse events after SAVR (23, 24), others demonstrated an increased risk of mortality, heart failure, and need for future mitral valve repair/replacement when mitral regurgitation was treated medically (2, 25, 26). While in some studies, mitral regurgitation did not emerge as independent predictor of mortality after TAVR (2, 20, 27), the majority of studies clearly pointed toward an increased risk of mortality when coexistent moderate to severe mitral regurgitation was present at the time of TAVR (28–34). In a meta-analysis including 4,839 TAVR patients, all-cause mortality was significantly higher in patients with moderate to severe mitral regurgitation (29). Similarly, in a multicenter registry including 1,007 patients undergoing TAVR with the CoreValve Revalving System, 1-year mortality was significantly higher in patients with moderate or severe mitral regurgitation as compared to those without (31). Differences in the grading methodology of mitral regurgitation which was based on qualitative echocardiographic measures in most studies, along with varying inclusion criteria, mainly regarding the etiology and severity of mitral regurgitation, may hamper comparisons among studies. Most interestingly, in the PARTNER (Placement of AoRTic TraNscathetER Valve) trial, patients with moderate to severe mitral regurgitation seemed to experience an even greater benefit from TAVR than those without, as reflected in a smaller number needed to treat to prevent a fatality (35).

## Treatment strategies in patients with severe aortic stenosis and mitral regurgitation

As double valve surgery is associated with an increased mortality as compared to SAVR or combined SAVR and coronary artery bypass grafting (36), transcatheter therapeutic options represent promising less-invasive treatment alternatives to open heart surgery in high-risk patients. Despite the high prevalence of concomitant mitral regurgitation in patients with severe aortic stenosis and the associated substantial morbidity and mortality, randomized trials investigating different therapeutic strategies are lacking. Whether concomitant mitral regurgitation should be treated medically or addressed in combined or staged procedures is ill-defined, and optimal patient selection and timing of interventions need to be determined. The evidence in this field is mostly stemming from observational data and case series, which precludes firm conclusions. Given the lack of guideline-based recommendations, personalized treatment strategies based on associated symptoms, the individual valvular pathology, the comorbid burden, and the estimated procedural risk are advocated (10). Irrespective of attempted surgical or transcatheter approaches to mitral regurgitation, guideline-based heart failure management is essential in these patients before evaluating the regurgitation severity.

### The guideline-based heart team approach

All patients with severe symptomatic aortic stenosis and concomitant mitral regurgitation, who are at increased surgical risk, are evaluated by a multidisciplinary Heart Team to ensure comprehensive risk stratification and optimal patient selection. Besides technical aspects, associated symptoms, the burden of comorbidities, patient's life expectancy, patient's frailty, and the quality of life need to be taken into account to deliver best quality of care (10). Thereby, a balanced decision on the optimal treatment strategy is taken for each individual patient.

Guideline-based indications for mitral valve procedures are summarized in Table 2. The distinction between primary and secondary mitral regurgitation is emphasized in this context. Although mitral valve repair/replacement is considered the gold standard in patients with symptomatic severe mitral regurgitation (10), benefits in those with secondary forms are less clear as lack of survival benefit and an increased risk of recurrence have been reported (37), finally resulting in lower levels of evidence for treatment recommendations in this patient subgroup. According to current guidelines of the European Society of Cardiology (ESC) (10), intervention for severe chronic primary mitral regurgitation is indicated in symptomatic patients with preserved left ventricular ejection fraction (LVEF >30%, class of recommendation I, level of evidence B) with valve repair being the preferred treatment approach. Surgery is further indicated in asymptomatic patients with left ventricular dysfunction as mirrored by a reduced left ventricular systolic function [LVEF ≤ 60%] or increased left ventricular dimensions (left ventricular end-systolic diameter ≥45 mm, class of recommendation I, level of evidence B), and should be considered in patients with new onset of atrial fibrillation or increased pulmonary pressures (systolic pulmonary pressure ≥50 mmHg, class of recommendation IIa, level of evidence B), and flail leaflet or significant left atrial dilatation (class of recommendation IIa, level of evidence C) (10). Percutaneous edge-to-edge repair may be considered by the Heart Team for symptomatic patients at high surgical risk (class of recommendation IIb, level of evidence C). Currently, there is no indication to intervene for moderate mitral regurgitation.

**Table 2:** Recommendations for the treatment of chronic mitral regurgitation according to the 2017 European Society of Cardiology (ESC) and European Association for Cardio-Thoracic Surgery (EACTS) guidelines for the management of valvular heart disease.

**Recommendations**	**Class of recommendation**	**Level of evidence**
**PRIMARY MITRAL REGURGITATION**		
**Mitral valve repair** is the treatment of choice when durable results are expected.	**I**	C
Mitral valve surgery is indicated in patients with **severe symptomatic** mitral regurgitation and preserved left ventricular systolic function (LVEF >30%).	**I**	B
Mitral valve surgery is indicated in asymptomatic patients with severe mitral regurgitation and left ventricular dysfunction **(LVEF** ≤ **60% or LVESD** ≥**45 mm)**.	**I**	B
Mitral valve surgery should be considered in asymptomatic patients with **atrial fibrillation** or **pulmonary hypertension** (systolic pulmonary pressure at rest >50 mmHg)	**IIa**	B
Mitral valve surgery should be considered in asymptomatic patients with low surgical risk, preserved left ventricular function (LVEF >60%) and LVESD between 40 and 44 mm, when durable repair is likely and there is a **flail leaflet** or **left atrial dilatation** (LAVI >60 ml/m2)	**IIa**	C
Mitral valve repair should be considered in **symptomatic** patients with low surgical risk and **severe left ventricular dysfunction** (LVEF <30% and/or LVESD >55 mm) refractory to optimal heart-failure therapy when **successful repair is likely**	**IIa**	C
Mitral valve replacement may be considered in **symptomatic** patients with low surgical risk and **severe left ventricular dysfunction** (LVEF <30% and/or LVESD >55 mm) refractory to optimal heart-failure therapy when **likelihood of repair is low**	**IIb**	C
**Percutaneous edge-to-edge repair** may be considered by the **Heart Team** in patients with symptomatic severe mitral regurgitation, who meet the echocardiographic criteria of eligibility and are deemed at **high or prohibitive surgical risk**	**IIb**	C
**SECONDARY MITRAL REGURGITATION**		
Mitral valve surgery is indicated in patients with **severe** mitral regurgitation undergoing **CABG**	**I**	C
Mitral valve surgery should be considered in patients with **severe symptomatic** mitral regurgitation and left ventricular dysfunction (**LVEF <30%**) with an option for **coronary revascularization**	**IIa**	C
Mitral valve surgery may be considered in patients with low surgical risk, preserved left ventricular systolic function (LVEF >30%) and **severe symptomatic** mitral regurgitation refractory to optimal heart-failure therapy	**IIb**	C
**Percutaneous edge-to-edge repair** may be considered in patients deemed at **high or prohibitive surgical risk** with no option for coronary revascularization, who have **severe symptomatic** mitral regurgitation refractory to optimal heart-failure therapy and meet the echocardiographic criteria of eligibility	**IIb**	C
**Percutaneous edge-to-edge repair or valve surgery** may be considered by the Heart Team in patients deemed at **high or prohibitive surgical risk** with no option for coronary revascularization and **severe left ventricular dysfunction** (LVEF <30%), who have **severe symptomatic** mitral regurgitation refractory to optimal heart-failure therapy and meet the echocardiographic criteria of eligibility	**IIb**	C

*Adapted from Baumgartner et al. (10). CABG, coronary artery bypass grafting; LAVI, left atrial volume index; LVEF, left ventricular ejection fraction; LVESD, left ventricular end-systolic diameter*.

In patients with severe secondary mitral regurgitation, optimal guideline-recommended heart failure therapy, including optimal medical therapy and coronary revascularization or cardiac resynchronization as indicated, is of particular importance (10, 38). For the treatment of severe secondary mitral regurgitation, a class I recommendation for mitral valve surgery with valve repair being the method of first choice exists in patients undergoing coronary artery bypass graft surgery, when LVEF is preserved (level of evidence C). In symptomatic patients with reduced left ventricular systolic function (LVEF <30%), surgery should be considered when coronary revascularization is indicated (class of recommendation IIa, level of evidence C). When there is no option for coronary revascularization, mitral valve surgery may be considered in patients with preserved LVEF and low surgical risk (class of recommendation IIb, level of evidence C). A percutaneous edge-to-edge procedure may be considered when echocardiographic criteria of eligibility are met and surgical risk deemed prohibitive (class of recommendation IIb, level of evidence C). Although transcatheter percutaneous mitral valve procedures were shown to substantially reduce the degree of mitral regurgitation, beneficially affect left ventricular reverse remodeling, and significantly decrease the symptomatic burden (15, 39–41), it remains uncertain whether survival benefits are achieved. Emerging interventional procedures such as transcatheter annuloplasty or transapical valve replacement complement the therapeutic armamentarium for severe mitral regurgitation in high risk patients. Experience with these procedures is, however, still limited and guideline-based recommendations lacking.

### TAVR and natural course of mitral regurgitation

Most studies report on a significant improvement of mitral regurgitation after AVR, which has mostly been attributed to reverse left ventricular remodeling and improved left ventricular function. Indeed, in a meta-analysis including 8,927 patients undergoing TAVR, the severity of mitral regurgitation significantly improved in about 60% of patients (42). In the PARTNER trial, moderate to severe mitral regurgitation was observed in 21% of SAVR and 20% of TAVR patients, and improvement was reported in 69% of SAVR and 58% of TAVR patients at 30 days (2). Similar results were reported in other studies (32, 43, 44). These effects may be particularly predominant in patients with secondary mitral regurgitation as structural valve alterations obviously persist after TAVR. A significant improvement in mitral regurgitation severity is more likely to occur in patients without severe pulmonary hypertension and atrial fibrillation (31) Interestingly, acute improvement in mitral regurgitation has been reported following TAVR and was related to immediate post-procedural changes in left ventricular hemodynamics and improved mitral leaflet tethering (45). Whether the design of the implanted transcatheter heart valve influences the post-procedural course of mitral regurgitation needs to be delineated in future studies. Observational studies point toward a greater degree of reduction of mitral regurgitation in patients treated with balloon-expandable as compared to self-expandable transcatheter heart valves (28).

### Combined TAVR and transcatheter mitral valve procedure

In comparison to a single valve procedure, surgical double valve replacement/repair is associated with increased morbidity and mortality (36, 46). Indeed, mortality rates of about 10% have been reported for double valve aortic and mitral surgery as compared to 3% for isolated SAVR (36). Over the last decades, transcatheter techniques have evolved and offer less-invasive treatment alternatives to double valve surgery in patients deemed at high or prohibitive surgical risk. Transcatheter mitral valve replacement (TMVR) offers a less invasive treatment alternative to redo cardiac surgery, particularly in high-risk patients with degenerated mitral bioprostheses and failed annuloplasty rings (47). Clinical experience with bivalvular transcatheter procedures, however, is still limited (48, 49). The success of a combined approach with transcatheter mitral valve repair performed at the time of TAVR has been reported in several studies (50, 51). Different transcatheter mitral valve repair technologies may be used in this context such as the MitraClip® device (Abbott Vascular Inc., Menlo Park, CA, USA), the Carillon Mitral Contour System® (Cardiac Dimensions, Kirkland, WA, USA), and the Cardioband® (Valtech, Edwards Lifescience Corp, Irvine, CA, USA) (52) An overview of current devices for transcatheter mitral valve repair is provided in Table 3.

**Table 3:** Overview of devices for transcatheter mitral valve repair.

**Device**	**Principle**	**Characteristics**
MitraClip (Abbot Vascular)	Edge-to-edge repair	- V-shaped clip is placed on the mitral valve leaflets via transseptal approach - Device produces a double mitral valve orifice
Pascal (Edwards Lifescience)	Edge-to-edge repair	- Central spacer with two paddles is placed on the mitral valve leaflets via transseptal approach - Device produces a double mitral valve orifice
Carillon (Cardiac Dimensions)	Indirect annuloplasty	- Anchors at both ends are connected by a curved nitinol ribbon connector - Device is implanted within the coronary sinus to decrease annular dimensions
Cardioband (Valtech, Edwards Lifescience)	Direct annuloplasty	- Annuloplasty band implanted around the posterior mitral annulus - Device decreases septolateral annular dimensions
Mitralign (Mitralign Inc.)	Direct annuloplasty	- Pledget delivery system with retrograde aortic access - Reduction of the annular circumference is achieved by two pairs of pledgets placed at opposite sites of the annulus and producing tissue plication
NeoChord DS 1000 (NeoChord Inc.)	Chordal repair	- Artificial chord-based system implanted via transapical access, secured to the leaflet and anchored to the left ventricular apex
Harpoon TSD-5 (Edwards Lifescience)	Chordal repair	- Artificial chord-based system implanted via transapical access, secured to the leaflet and anchored to the left ventricular apex

**Figure 1:**
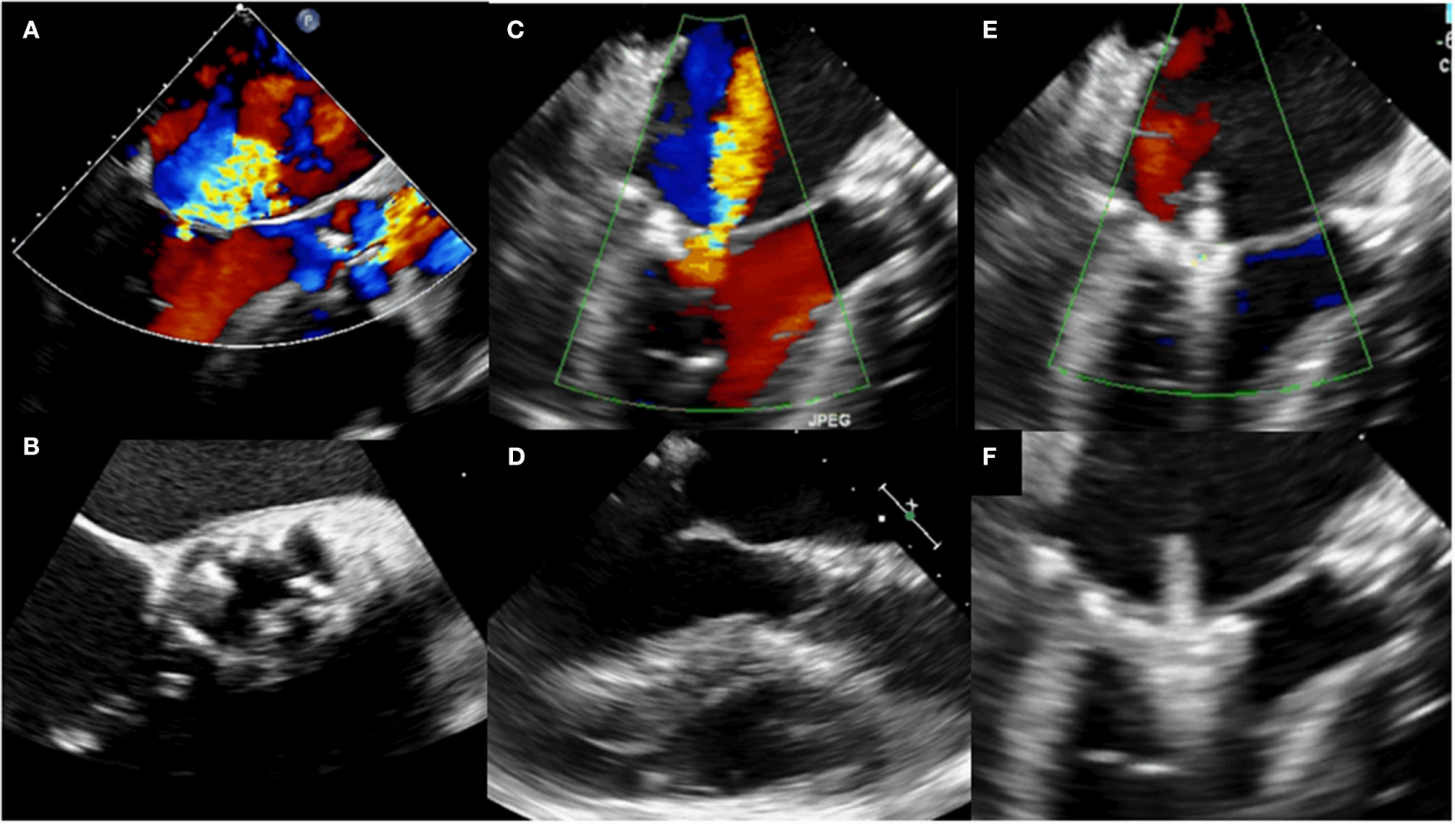
Pre- and post-procedural transesophageal echocardiography in a patient undergoing staged transcatheter aortic valve replacement (TAVR) and edge-to-edge mitral valve repair with the MitraClip® system. **(A)** Transesophageal color Doppler echocardiography at baseline (three-chamber view) showing severe aortic stenosis and concomitant severe mitral regurgitation. **(B)** Transesophageal echocardiography at baseline (aortic valve short-axis view) showing severe aortic stenosis. **(C)** Two-dimensional transesophageal color Doppler echocardiography (three chamber view) showing persistent severe mitral regurgitation following TAVR. **(D)** Two-dimensional transesophageal echocardiography (three chamber view) following TAVR. **(E)** Two-dimensional transesophageal color Doppler echocardiography (three-chamber view) during staged percutaneous edge-to-edge mitral valve repair with the MitraClip® system (grasping). **(F)** Two-dimensional transesophageal echocardiography (three-chamber view) during staged percutaneous edge-to-edge mitral valve repair with the MitraClip® system (grasping).

The most advanced percutaneous mitral valve repair system is the MitraClip® device which allows for introducing a V-shaped clip on the mitral valve leaflets via a transseptal approach under transesophageal echocardiographic and fluoroscopic guidance (Figure 1). Thereby, a double or multiple orifice is created (14–16). High procedural success rates of percutaneous edge-to-edge mitral valve repair have not only been reported for primary, but also secondary mitral regurgitation (41, 53), and safety and efficacy was also demonstrated in patients who did not meet the key echocardiographic eligibility criteria as determined by the EVEREST (Endovascular Valve Edge-to-Edge Repair) studies (54).

Percutaneous indirect mitral annuloplasty was developed to improve leaflet coaptation by reducing mitral annular dimensions using dedicated transcatheter devices such as the Carillon Mitral Contour System®. The Carillon Mitral Contour System® consists of anchors at both ends, which are connected by a curved nitinol ribbon connector. The device is implanted within the coronary sinus to reduce the severity of mitral regurgitation by annular placation (14, 55). Safety and feasibility of the procedure, along with clinical benefits in terms of heart failure symptoms, quality of life, and exercise tolerance have been shown for patients with dilated cardiomyopathy and functional mitral regurgitation in different studies such as the AMADEUS (the Carillon Mitral Annuloplasty Device European Union Study) and the TITAN (Transcatheter Implantation of Carillon Mitral Annuloplasty Device) trials (40, 55). The direct annuloplasty Cardioband® system represents a similar interventional transseptal approach for the treatment of secondary mitral regurgitation (56) The annuloplasty band is implanted around the posterior mitral annulus, aiming at reducing mitral regurgitation by decreasing septolateral annular dimensions.

Besides minimally invasive surgical valve repair or replacement, TMVR has emerged as less-invasive treatment alternative for patients deemed at high or prohibitive surgical risk, with several prostheses already introduced in clinical practice (57–59). Although feasibility and safety of valve-in-valve, valve-in-ring, and valve-in-native ring procedures have been demonstrated for transcatheter heart valve implantation in the mitral position (60), future randomized studies are needed to determine the role of TMVR in patients with severe mitral regurgitation.

### TAVR and staged transcatheter mitral valve procedure

As significant improvements in mitral regurgitation severity have been observed after AVR (26, 43, 44), a staged approach may be favored over a combined procedure with the aortic valve being addressed first and the mitral valve treated only in patients who remain symptomatic in spite of successful TAVR (50, 61). Patients with prior AVR undergoing transcatheter mitral valve repair, however, represent a complex patient subgroup with a high comorbid burden at increased risk of adverse events. One-year survival in these patients was reported to be below 50% (62).

Given the lack of randomized comparisons between surgical and transcatheter double valve interventions vs. medical management of mitral regurgitation in the context of severe aortic stenosis, evidence-based recommendations on patient selection and optimal timing of interventions cannot be made. For predominantly secondary mitral regurgitation, when no major structural mitral valve defects exist, a staged approach may be reasonable to tailor mitral interventions to patients with persistent symptomatic mitral regurgitation, who may benefit most. Bivalvular interventions may be advocated when concomitant predominantly primary mitral regurgitation is present.

Based on our experience, we strongly advocate a stepwise approach in this high-risk patient population, with TAVR being performed first and percutaneous mitral valve repair considered by the Heart Team only when severe mitral regurgitation persists after TAVR. A close clinical and echocardiographic follow-up of these patients following TAVR is mandatory, with functional tests used when grading of mitral regurgitation is challenging.

### Cost-effectiveness of transcatheter valve procedures

Although procedural costs of TAVR exceed those of SAVR, cost-effectiveness of TAVR in patients at increased surgical risk has been demonstrated when shorter hospital stay and reduced need for post-acute rehabilitation services are taken into account, particularly when a transfemoral access is suitable (63–66). In heart failure patients with moderate-to-severe mitral regurgitation, therapy with the MitraClip® device was shown to be cost-effective compared to medical management alone (67). Direct economic comparisons between different transcatheter mitral valve repair systems and mitral valve surgery are, however, lacking. A staged approach with TAVR performed first and percutaneous mitral valve repair tailored to patients who do not experience any improvement in mitral regurgitation following TAVR seems to be cost-effective, as thereby the number of mitral valve interventions is reduced in comparison to simultaneous procedures.

## Conclusion

Risk assessment and optimal patient selection, along with a personalized treatment approach defined by the Heart Team, is important to ensure best patient care in symptomatic aortic stenosis and concomitant mitral regurgitation. Given the heterogeneity and complexity of mitral valve disease in these high-risk patients, individualized treatment concepts are needed. Although the feasibility and safety of bivalvular transcatheter procedures have been demonstrated, the treatment of first choice—TAVR only, staged TAVR and transcatheter mitral valve procedures, or combined bivalvular transcatheter therapy vs. minimally-invasive surgical treatment—remains to be determined. Randomized trials investigating benefits of mitral valve procedures vs. guideline-based heart failure therapy in TAVR patients with concomitant mitral regurgitation will help to define optimal treatment approaches. Refinements of transcatheter mitral valve concepts including the combination of different approaches will probably enter clinical practice in near future and further improve patient outcomes.

## Author contributions

BS analysis and interpretation of the literature, drafting of the manuscript. BS, MR, DL, UL, and AL revising the manuscript critically for important intellectual content, final approval of the manuscript submitted. AL analysis and interpretation of the literature.

### Conflict of interest statement

The authors declare that the research was conducted in the absence of any commercial or financial relationships that could be construed as a potential conflict of interest.
